# Unicentric mesenteric Castleman’s disease with littoral cell angioma, anemia, growth retardation and amenorrhea: A case report

**DOI:** 10.3892/ol.2015.2933

**Published:** 2015-02-05

**Authors:** LING YANG, SHUANG ZHAO, RONG-BO LIU

**Affiliations:** Department of Radiology, West China Hospital, Sichuan University, Chengdu, Sichuan 610000, P.R. China

**Keywords:** Castleman’s disease, littoral cell angioma, anemia, computed tomography, growth retardation

## Abstract

Castleman’s disease (CD) is a rare lymphoproliferative disorder of unknown origin, and littoral cell angioma (LCA) is a rare vascular tumor of the spleen with an unknown etiology. The current study reports the case of a 28-year-old female who presented with anemia, growth retardation and amenorrhea. Physical examination revealed a mass in the mesentery, splenomegaly with multiple small nodules, hepatomegaly and an infantile uterus. Histopathological analysis of the resected mass and spleen confirmed the diagnosis of hyaline-vascular CD and LCA. The patient’s anemia resolved, and menstruation and breast development also commenced following surgery. To the best of our knowledge, this is the first report of CD accompanied by littoral cell angioma, anemia, growth retardation and amenorrhea.

## Introduction

Castleman’s disease (CD) is a rare lymphoproliferative disorder with unknown origin. It is not frequently identified in the abdomen, however, it can occur in any region containing lymph nodes (1,2). Two principal histological subtypes of CD have been reported: Hyaline-vascular and plasma cell variants; additionally, there are two clinicoradiological entities, denoted unicentric and multicentric CD (1). CD is exclusively diagnosed histologically and immunohistochemically following biopsy or resectioning of the lesion. Furthermore, the majority of unicentric cases are cured by resection of the involved lymph node(s). Radiation or chemotherapy may be administered, however, these treatments are not curative (1,3). The five-year survival rate for unicentric CD is ~100% (3).

Littoral cell angioma (LCA) is also a rare disease, and refers to a benign, vascular tumor of the spleen (4), although malignant variants have been described (5,6). Although no age predilection has been identified, the median age for patients with LCA is 50 years (range, 1–77 years) and it usually occurs in adults and appears to be extremely rare in children. Currently, a final diagnosis is only possible by histopathological examination. LCA may be cured by with a splenectomy (5,6).

The current study describes the clinical, laboratory, radiological and histological findings, and the successful treatment of a patient with concurrent CD and LCA. Written informed consent was obtained from the patient. To date, no cases of CD accompanied by LCA have been previously reported.

## Case report

A 28-year-old female presented to the Department of Radiology, West China Hospital (Chengdu, China) with growth retardation, primary amenorrhea and a 10 year history of microcytic hypochromic anemia that was resistant to iron therapy. The patient had long-term paleness and gradually reduced physical strength. On physical examination, the patient was 145 cm tall and exhibited no secondary gender characteristics. In addition, moderate hepatomegaly, splenomegaly and a nontender mass in the hypogastrium were observed.

**Figure 1:**
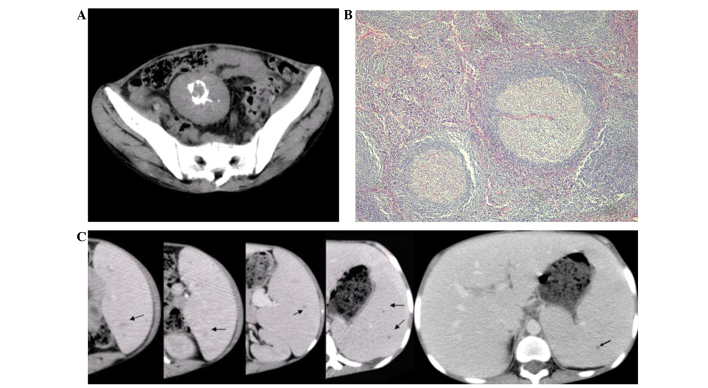
(A) A well-demarcated, moderately enhanced mass with ring-shaped calcification in the center was detected in the mesentery. (B) Hematoxylin and eosin-stained section of the mesentery mass showed involuting germinal center surrounded by concentric rings of small lymphocytes penetrated by hyalinized vessel (original magnification, ×200). (C) Enhanced abdominal computed scan on portal venous phase showed hepatomegaly and splenomegaly with scattered small low-attenuated lesions (arrows showing the lesions of littoral cell angioma in the spleen).

Laboratory test results indicated microcytic hypochromic anemia [hemoglobin, 14 g/l (normal range, 110–150 g/l); hematocrit, 7% (normal range, 35–45%); and mean corpuscular volume, 70.0 fl (normal range, 80–98 fl)], and thrombocytosis (396×10^9^/l; normal range, 100–300×10^9^/l), however, leukocyte count and hemoglobin electrophoresis were normal. A bone marrow smear revealed increased erythrocytic series (41%; normal range, 20–25%) and decreased granulocytic series (46.5%; normal range, 50–60%). Hormone, tumor marker, hepatic and renal function test results were all within normal ranges. An infantile uterus and a solid mass with central calcification were detected by pelvic ultrasonic examination. A moderately enhanced, well-defined mass of 4.8×5.6 cm in size was detected at the mesentery on computed tomography (CT) imaging; centrally located ring-shaped calcification could also be observed. In addition, hepatomegaly and splenomegaly were detected. Scattered small hypo-attenuated nodules were identified in the spleen on the arterial and portal venous phases of enhanced CT images, however, these were isoattenuated on normal CT images (Fig. 1A and C). No abnormalities were observed on liver parenchyma, chest or cervical CT imaging.

As the underlying nature of the mass, nodules of the spleen and hepatomegaly could not be determined in this patient, exploratory laparotomy was performed to remove the mass and spleen, with additional liver biopsy. Subsequent histopathological analysis of the mass was consistent with hyaline-vascular CD, revealing involuting germinal centers surrounded by concentric rings of small lymphocytes penetrated by hyalinized vessels (Fig. 1B). Immunohistochemically, the mass was reactive for CD3±, CD20±, bcl-2 (+) and Ki-67. In addition, pathological and immunohistochemical examination of the spleen showed a hybrid endothelial-histiocytic phenotype, which confirmed a diagnosis of littoral-cell angioma. Fatty degeneration was identified in the specimen of the liver. Following surgery, the patient’s symptoms resolved and laboratory tests normalized. Follow-up was performed once a month, and menstruation and breast development commenced three months following the surgery. At the time of writing, no recurrence had been identified.

## Discussion

CD was initially first described in 1954 and is also known as angiofollicular hyperplasia or giant lymph node hyperplasia; it is a rare disorder of lymphoid tissue (7). Little is known about the cause of this disorder, however, it is thought to be infectious, inflammatory, or hamartomatous in nature (1). CD may occur in any area in which lymphoid tissue is normally found, however, it most commonly occurs in the mediastinum (70%). Additionally, extrathoracic sites have been reported in the neck, axilla, mesentery, pelvis, pancreas, adrenal, and retroperitoneum (2).

Clinically, the hyaline-vascular type of CD is typically asymptomatic and tends to be localized, whereas the less common plasma cell variant is occasionally associated with symptoms including fever, anemia, weight loss, night sweats, and polyclonal hypergammaglobulinemia, and is typically multicentric (2). The present case exhibited unique clinical features including amenorrhea, growth retardation and anemia. The mechanism for growth retardation in CD is unclear, and multiple factors may be related to this, with IL-6 being one of the important contributing factors (8). We hypothesize that amenorrhea, which is extremely rare in CD (9), may be associated with the infantile uterus resulting from growth retardation. Anemia is also extremely rare in the hyaline-vascular CD, and Yang *et al* (8) proposed that elevated IL-6 acts on the IL-6 receptors in liver and tumor lymph node cells, resulting in the enhanced expression of hepcidin, consistent with the role of the IL-6/hepcidin pathway in anemia of chronic disease in CD (9).

LCA, initially described in 1991, is a rare vascular tumor, originating from the splenic littoral cells (10). Due to the limited number of cases, the etiology of LCA remains unclear. The histopathological characteristics of LCA include the presence of anastomosing vascular channels lined with flat and tall endothelial cells, with focal papillary fronds extended into the vascular channels and normal splenic sinuses at the periphery of the lesions in the splenic red pulp. Immunohistochemical analysis also exhibits specific staining patterns, as the tumor shows immunoreactivity with factor 8, CD31 and CD68, which indicates the presence of endothelial and histocytic cells (4).

Clinically, patients with LCA may present with an abdominal mass from splenomegaly or as an incidental finding. Symptoms of hypersplenism (thrombocytopenia and anemia), portal hypertension and pyrexia of unknown origin have also been described, but are rare (5). An association between LCA and malignancy, chronic infection or autoimmune diseases has been proposed (6), however, the coexistence of LCA and CD has not been previously reported in the literature. The association between CD and LCA in the present case was unclear, however, they have previously exhibited an association with altered immune host response (6) thus, we hypothesized that the coexistence of these two rare diseases in one patient may be due to immune disorder.

Unicentric CD exhibits a number of important features on imaging. It is usually shown to be a single, well circumscribed, soft-tissue mass with moderate to intense enhancement on CT, and may be homogeneous or heterogeneous dependent on its size; additionally, low attenuation areas in the mass has been shown to correspond to necrosis (11,12). Intratumoral calcification, found in approximately 5–10% of CD cases, is considered to be another characteristic CT feature of this disease (11). CD typically displays a variety of patterns in the abdomen and pelvis, including punctuate, coarse, flocculent, peripheral and arborizing pattern, which radiates from the center of the mass (2,12); however, the central ring-shaped pattern observed in the present case has not been previously described.

Conversely, the CT features of LCA have been well described, with the majority being isoattenuating to slightly hypoattenuating on non-contrast examinations. On contrast enhancement, early hypoattenuation on arterial and most early portal phase scans may be revealed. Heterogeneous to homogeneous enhancement is present on the late portal phase and delayed phase. However, a number of delayed scans have revealed a complete contrast washout with return to isoattenuation (13). In the current study, the patient exhibited a significantly enlarged spleen with scattered small hypoattenuated nodules on enhanced CT, which was imperceptible on plain CT images.

Surgical resection of the mass is usually curative in localized CD, with dissipation of the symptoms, and splenectomy is curative in littoral-cell angioma. However, local recurrence following subtotal resection has also been described in localized CD (1), as well as long-term complications with malignancy, which has been reported in localized CD and LCA (1,6), therefore, close clinical follow-up is required.

In conclusion, the present study reported an unusual case of unicentric CD accompanied by LCA, anemia, growth retardation and amenorrhea, which was treated with surgery. However, the relationship between unicentric CD and LCA requires further investigation.
